# Inhibition of Toll-like Receptor-4 expression for amelioration of myocardial injury in diabetes: A meta-analysis

**DOI:** 10.1016/j.clinsp.2022.100137

**Published:** 2022-11-23

**Authors:** Jinxin Yuan, Xingwen Yin, Hua Jiang

**Affiliations:** The Second Affiliated Hospital of Dalian Medical University, Liaoning, China

**Keywords:** Diabetic cardiomyopathy, Myocardial injury, TLR4, Meta-analysis

## Abstract

To understand the relationship between the inhibition of Toll-Like Receptor-4 (TLR4) expression levels and diabetic myocardial injury, studies on TLR4 and diabetic myocardial injury in the China National Knowledge Internet (CNKI), WanFang database, VIP Database, PubMed, The Cochrane Library, Web of Science, and other databases were explored (retrieval details: November 2020). A meta-analysis of the selected literature was performed using the RevMan 5.4 software to detect publication bias using funnel plots and conduct a sensitivity analysis. Nine publications were finally included in this study, of which six included data on Heart Weight/Body Weight (HW/BW) indexes, and five included data on Left Ventricular Systolic Pressure (LVSP) and Left Ventricular End-Diastolic Pressure (LVEDP) indices. The meta-analysis showed that HW/BW was significantly reduced after the suppression of TLR4 expression (Standardized Mean Difference [SMD = 1.9], 95% CI between 0.59 and 3.21, p = 0.004), LVSP was significantly improved (SMD = -2.39, 95% CI between -4.32 and -0.46, p = 0.02), and LVEDP was significantly reduced (SMD = 2.88, 95% CI between 1.05 and 4.71, p = 0.002). The TLR4 signaling pathway plays an essential role in the pathogenesis of Diabetic Cardiomyopathy (DCM). Inhibition of TLR4 expression can improve the degree of cardiac impairment. TLR4 may become a new target for the treatment of DCM, and the use of TLR4 inhibitors may prove to be a novel strategy for therapeutic research.

## Highlights

• A meta-analysis was applied to understand the relationship between inhibition of Toll-like Receptor 4 and diabetic myocardial injury.
• Inhibition of Toll-like Receptor 4 expression levels can improve the degree of cardiac impairment.
• Toll-like Receptor 4 may become a new target for the treatment of diabetic cardiomyopathy.

## Introduction

Diabetic Cardiomyopathy (DCM) is a myocardial disease in patients with diabetes and cannot be explained by hypertensive heart disease, coronary atherosclerotic heart disease, or other cardiac lesions. Based on the development of metabolic disorders and microvascular lesions, this disease results in multifocal myocardial necrosis and subclinical cardiac dysfunction and eventually progresses to heart failure, arrhythmia, and cardiogenic shock, leading to sudden death in patients with increased disease severity. In 1972, Rubler et al.^1^ autopsies of four patients with heart failure in diabetic glomerulosclerosis and found that these patients presented with no apparent etiology of heart failure other than the presence of diabetes mellitus. Thus, the concept of “diabetic cardiomyopathy” was introduced. The structural changes in DCM were characterized based on observed manifestations, such as near-normal left ventricular end-diastolic volume, increased left ventricular weight and wall thickness, myocardial hypertrophy and fibrosis, and fatty deposits in cardiomyocytes. Functional changes are characterized by impaired diastolic function without significant systolic function impairment and reduced ventricular wall elasticity.^2^

Zhang et al.^3^ demonstrated that cardiac apoptosis in diabetic mice could be prevented by silencing TLR4 gene expression. Jiang et al. found that mice with TLR4 deletion manifested suppressed symptoms of myocardial hypertrophy induced by aortic constriction.^4^ Numerous studies have shown that the TLR4/NF-κB signaling pathway is closely associated with the development of cardiovascular disease, and the TLR4 protein can enter the nucleus via the intracellular transport pathway, thereby leading to the activation of NF-κB and release induction of substantial amounts of inflammatory factors from cells, resulting in the development of an inflammatory response that causes myocardial cell necrosis, increased myocardial infarct size, and reduced cardiac function.^5^, ^6^, ^7^, ^8^

Therefore, in this study, the authors used meta-analysis to observe the extent of myocardial injury in diabetic rats after the inhibition of TLR4 expression levels to provide evidence-based medical information for the treatment and prevention of diabetic cardiomyopathy.

## Materials and methods

### Literature inclusion and exclusion criteria

The inclusion criteria were as follows: 1) The study was a Randomized Controlled Trial (RCT) with Chinese and English literature. 2) Diabetic rats were successfully used as models for the study subjects. 3) Intervention: The treatment group was administered drugs to inhibit TLR4 expression, and the TLR4 expression was significantly different from that in the model group (p < 0.05). 4) Outcome indicators: a) Tumor Necrosis Factor-α (TNF-α) levels; b) Left Ventricular Systolic Pressure (LVSP); c) Left Heart End-Diastolic Pressure (LVEDP); d) Cardiac weight index (Heart Weight/Body Weight, HW/BW); and e) Left ventricular weight index (Left Ventricular Weight/Body Weight, LVW/BW).

Exclusion criteria: 1) Reviews and conference papers; 2) Case reports and duplicate publications; 3) Literature with incomplete data and inaccessible full text; 4) Literature that did not meet the inclusion criteria indicators or did not contain any of the outcome indicators; 5) Outcome indicators did not contain available specific values (mean ± standard deviation), with only images presenting the results; and 6) Research models that included human subjects, mice, and cell lines (Table 1).

**Table 1:** Chinese search strategy.

Chinese database	Data retrieval strategy	Results
China Knowledge Network Full Text Database (CNKI)	(SU = ('TLR4′ + 'toll-like receptor 4′) AND SU = ('hyperglycemia' + 'diabetes mellitus') AND SU = ('myocardial apoptosis' + 'cardiomyopathy')) OR (SU = ('TLR4′ + 'toll-like receptor 4′) AND SU = (DC + DCM + 'diabetic cardiomyopathy'))	33 articles
Wanfang Database	Subject: (("TLR4" or "toll-like receptor 4") and ("hyperglycemia" or "diabetes") and ("myocardial apoptosis" or "cardiomyopathy")) or (("TLR4" or "toll-like receptor 4") and (DC or DCM or "diabetic cardiomyopathy "))	294 articles
VIP Database	((M = "TLR4" OR "toll-like receptor 4") AND (M = "hyperglycemia" OR "diabetes") AND (M = "myocardial apoptosis" OR "cardiomyopathy")) OR ((M = "TLR4" OR "toll-like receptor 4") AND (M = DC + DCM + "diabetic cardiomyopathy"))	28 articles

### Literature retrieval method

**Fig. 1:**
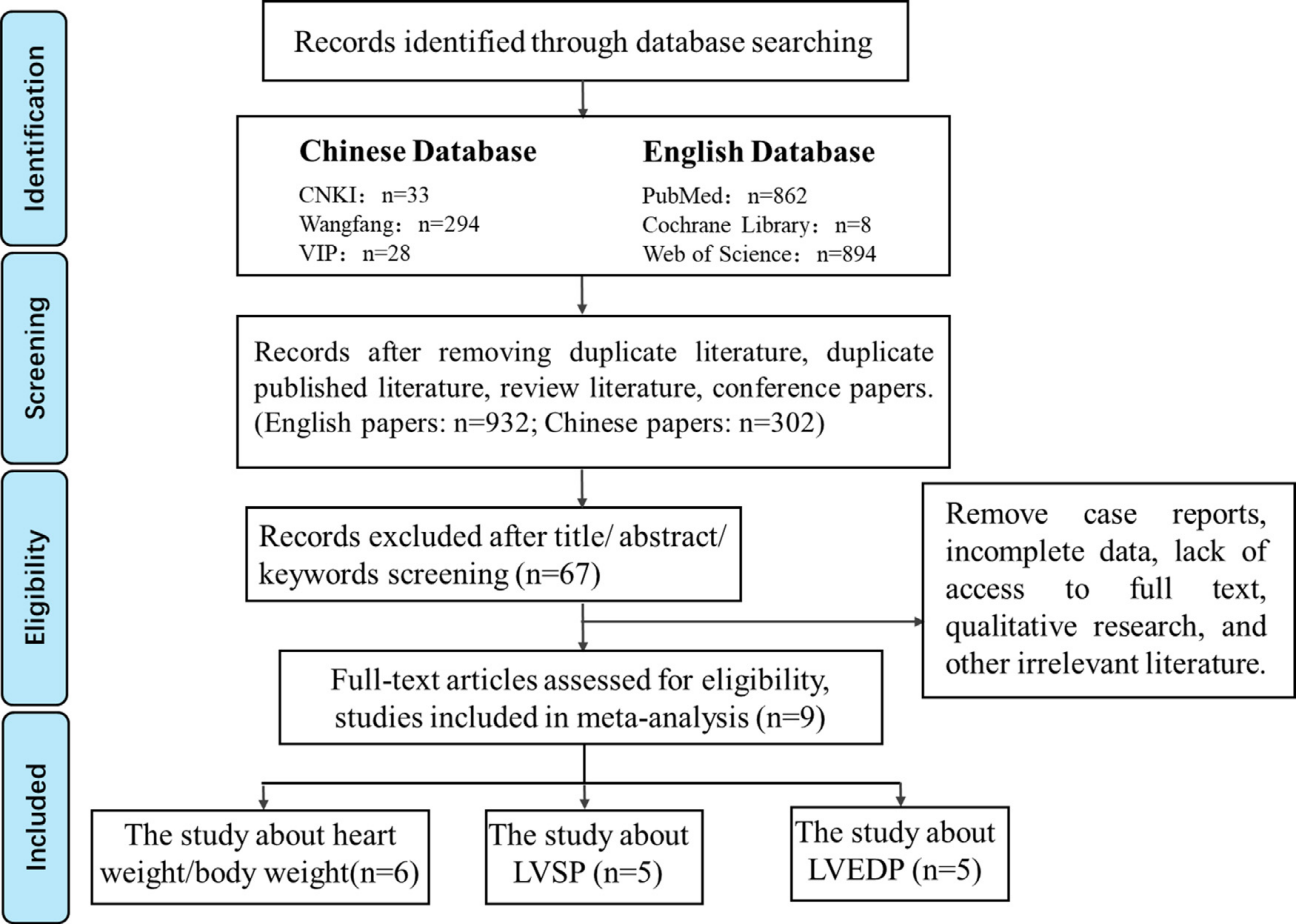
Literature screening and the results obtained.

From October 2020 to November 2020, literature retrieval was conducted using the China National Knowledge Infrastructure (CNKI), WanFang Database (WanFang Data), VIP Database, PubMed, Cochrane Library, and Web of Science databases. The search strategy and preliminary search results using Chinese databases (referred to as the Chinese search strategy) are shown in Fig. 1; the search strategy and preliminary search results using English databases (referred to as the English search strategy) are shown in Table 2.

**Table 2:** English search strategy.

English database	Retrieval	Results
PubMed	((TLR4 [Title/Abstract] or Toll-like receptor-4 [Title/Abstract]) and (hyperglycemia [Title/Abstract] or hyperglycemia [Title/Abstract] or high blood glucose or diabetes [Title/Abstract] or diabetes mellitus [Title/Abstract]) and (myocardial apoptosis [Title/Abstract] or myocyte apoptosis [Title/Abstract] or myocardiopathy [Title/Abstract] OR cardiomyopathy [Title/Abstract] or Cardiomyopathies [Title/Abstract])) OR ((TLR4 [Title/Abstract] OR Toll-like receptor-4 [Title/Abstract])AND (diabetic cardiomyopathy [Title/Abstract] OR Diabetic Cardiomyopathies [Title/Abstract] or DCM [Title/Abstract] or DC [Title/Abstract]))	862 articles
The Cochrane Library	(((TLR4):ti,ab,kw OR (Toll-like receptor-4): ti,ab,kw) AND ((hyperglycemia):ti,ab,kw OR (hyperglycemia):ti,ab,kw OR (high blood glucose):ti,ab,kw OR (diabetes mellitus):ti,ab,kw OR (diabet*):ti,ab,kw) AND ((myocardial apoptosis):ti,ab,kw OR (myocyte poptosis):ti,ab,kw OR (myocardiopathy):ti,ab,kw OR (cardiomyopathy*):ti,ab,kw)) OR (((TLR4):ti,ab,kw OR (Toll-like receptor-4): ti,ab,kw) AND ((diabetic cardiomyopathy):ti,ab,kw OR (diabetic cardiomyopathies):ti,ab,kw OR (DCM):ti,ab,kw OR (DC):ti,ab,kw))	8 articles
Web of Science	(TS = (TLR4 or Toll-like receptor-4) and TS = (hyperglycemia or hyperglycemia or high blood glucose or diabetict* or diabetes mellitus) and TS = (myocardial apoptosis or myocyte apoptosis or myocardiopathy or cardiomyopathy or cardiomyopathies)) or (TS= (TLR4 or Toll-like receptor-4) and TS = (diabetic cardiomyopathy or diabetic cardiomyopathies or DCM or DC))	894 articles

### Literature screening and data extraction

Two researchers explored the published articles independently based on the retrieval strategy, and the data on duplicated literature, republished literature, review literature, and conference papers were removed. Based on the preliminary filtered dataset, the two researchers read the titles, abstracts, and keywords separately and assessed the suitability of their inclusion based on the inclusion criteria to obtain the secondary filtered dataset. The full text of the literature in the secondary screening dataset was downloaded and read to exclude literature that did not meet the inclusion criteria. If a dispute occurred, it was resolved through discussion with a third party to reach a consensus. For literature with unidentified data, the necessary information was collected as much as possible by contacting the first author or the corresponding author.

Information extracted from the literature included: 1) Basic information about the study, first author, year of publication, article title, experimental animal species, modeling method, and sample size; 2) Interventions, duration of intervention, and intervention drugs, 3) Key elements of risk of bias assessment, and 4) Outcome indicators and result measurement data.

### Risk of bias assessment of the included studies

The risk of bias assessment of the included literature was evaluated independently by two researchers, and disputes were resolved in consultation with a third party. The quality of the included studies was evaluated using the risk of bias assessment tool recommended in the Cochrane Handbook for Systematic Reviews of Interventions. A total of seven parameters were included to assess the risk of bias, comprising six aspects of selection (including random sequence generation and allocation concealment), implementation (including subjecting personnel and participants to blinded studies), measurement (blinded evaluation of study outcomes), follow-up (completeness of outcome data), reporting (selective reporting of study results), and other (other sources of bias). For each parameter, “low risk of bias”, “high risk of bias”, and “unclear risk of bias” were determined based on the risk of bias assessment guidelines.

### Statistical analysis

Meta-analysis was performed using the RevMan 5.4 software data from the included literature, comprising continuous variables. The Standardized Mean Difference (SMD) was used as the effect index with each effect, their point estimates, and a 95% CI. Analysis of the heterogeneity between the included studies was performed using the χ2 test, while the magnitude of heterogeneity was determined quantitatively with I^2^. Meta-analysis was performed using a fixed-effects model if the heterogeneity among the results was not significant (I^2^ ≤ 50%). A random-effects model was used if the heterogeneity was significant among the studies (I^2^ > 50%). Meta-analysis results were mainly presented as forest plots, and funnel plots were used to analyze the presence of publication bias; sensitivity analysis was also performed.

## Results

### Basic information of the included literature

#### Literature screening

In this study, 355 Chinese and 1764 English publications were initially retrieved, 67 publications were obtained after the secondary screening, and 9 publications were finally included after the tertiary screening, as shown in Fig. 1. Six studies focused on HW/BW, five focused on LVSP, and five focused on LVEDP.

#### Basic characteristics of the included literature

A total of nine publications were included in this meta-analysis, seven of which were written in Chinese and two in English. The detailed characteristics of the included studies are listed in Table 3.

**Table 3:** Basic characteristics of the included studies.

First author	Rat characteristics	Modeling method	Group division	Sample size	Interventions	Duration of intervention	Outcome indicators^a^
Shi Hui 2019	Male SD rats 160∼200 g	High-fat feed combined with a single intraperitoneal injection of STZ 35 mg/kg	Model group	8	Distilled water 20 mg/(kg-d)	8 weeks	4, 5
			Treatment group	8	Danchuang hypoglycemic capsule 20 mg/(kg-d)	8 weeks	4, 5
Jin Baolan 2017	Male Wistar rats 190∼220g	Single intraperitoneal injection of STZ 55 mg/kg	Model group	10	Distilled water 16.7 mg/(kg-d)	6 weeks	2, 3
			Treatment group	10	Detoxification and Tongluo Infusion 16.7 g/(kg-d)	6 weeks	2, 3
Zhang Hongli 2017	Male Wistar rats 120–160g	Single intraperitoneal injection of STZ 70 mg/kg	Model group	10	Physiological saline 8 mL/d	6 weeks	2, 3, 4
			Treatment group	10	Ginseng and peony oral solution 8 mL/d	6 weeks	2, 3, 4
Guo Xin 2016	Male SD rats 160∼170 g	High-fat feed combined with a single intraperitoneal injection of STZ 30 mg/kg	Model group	6	Physiological saline 200 μg/(kg-d)	12 weeks	4
			Treatment group	6	Leucoxanthin 200 μg/(kg-d)	12 weeks	4
Dai Yannan 2016	Male Wistar rats 140-‒180g	High-fat feed combined with a single intraperitoneal injection of STZ 25 mg/kg	Model group	8	Physiological saline 250 mg/(kg-d)	8 weeks	2, 3
			Treatment group	9	Ginseng and Peony Oral Liquid 250 mg/(kg-d)	8 weeks	2, 3
Zheng Zhenzhong 2015	Male SD rats 200∼220g	Single intraperitoneal injection of STZ 65 mg/kg	Model group	8	/	12 weeks	1, 4, 6
			Treatment group	8	Silencing of the Fgl2 gene	12 weeks	1, 4, 6
Chen Tingting 2015	Male SD rats 180∼220g	Single intraperitoneal injection of STZ 35 mg/kg	Model group	7	Physiological saline 250 mg/(kg-d)	12 weeks	2, 3
			Treatment group	7	Flavoprotein 250 mg/(kg-d)	12 weeks	2, 3
Zhong Yuan 2012	Male SD rats 180∼220g	Single intraperitoneal injection of STZ 55 mg/kg	Model group	18	Physiological saline 20 mg/(kg-d)	12 weeks	4
			Treatment group	18	Simvastatin group 10 mg/(kg-d)	12 weeks	4
Zhu Xiaoying 2009	Male GK rats 242–294 g	/	Model group	8	Distilled water 50 mg/(kg-d)	5 weeks	1, 2, 3, 4
			Treatment group	8	Atorvastatin 50 mg/(kg-d)	5 weeks	1, 2, 3, 4

a Outcome indicators: (1) Tumor Necrosis Factor-α (TNF-α) level, (2) Left Ventricular Systolic Pressure (LVSP), (3) Left Heart End-Diastolic Pressure (LVEDP), (4) Cardiac Weight Index (Heart Weight/Body Weight, HW/BW); (5) Left Ventricular Weigh Index (Left Ventricular Weight/Body Weight, LVW/BW).

#### Results of the risk of bias assessment for the included literature

**Fig. 2:**
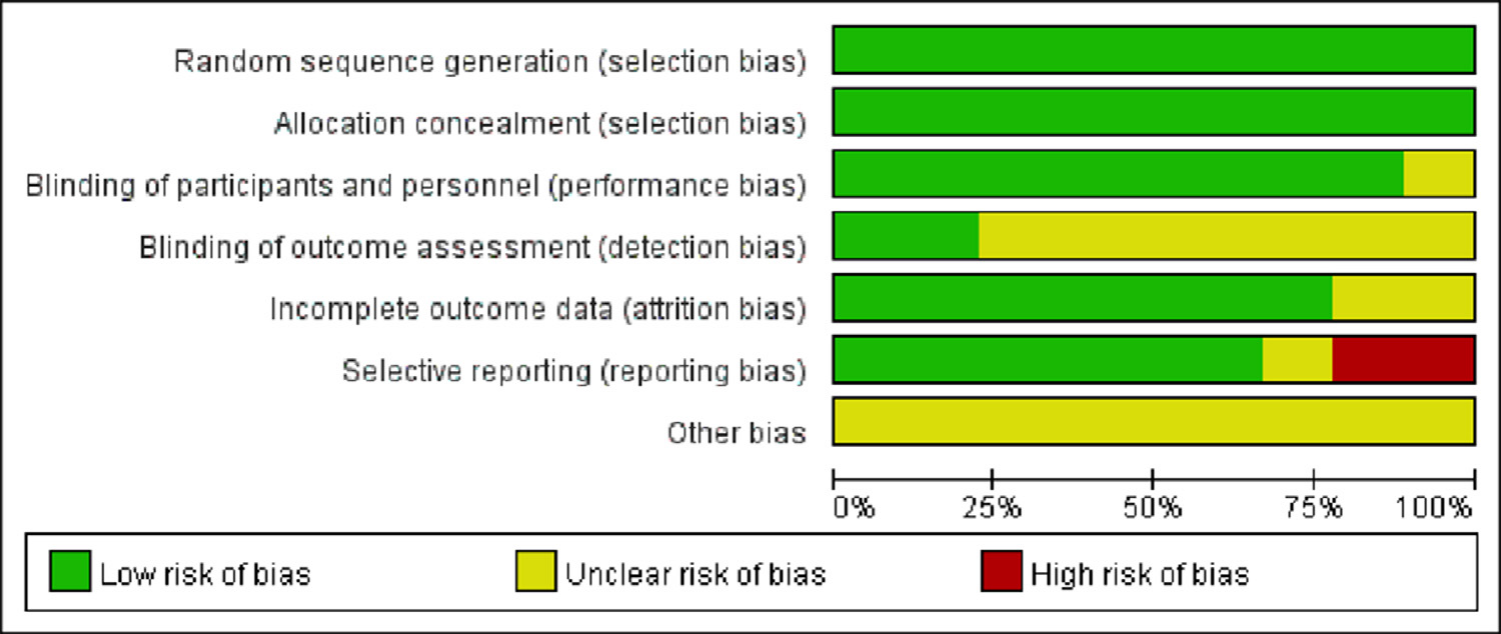
Graph of the risk of bias for the included literature.

**Fig. 3:**
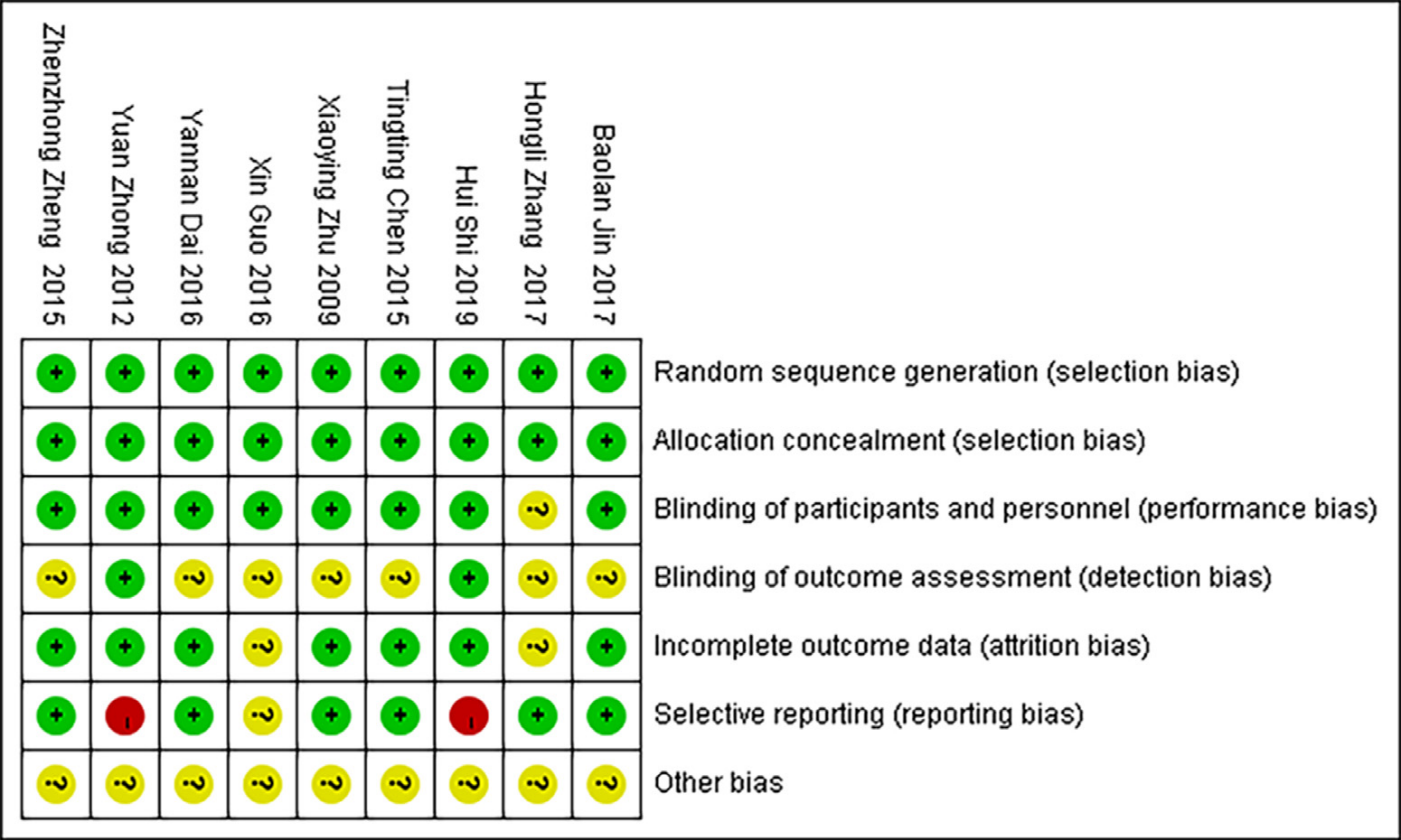
Summary graph showing the risk of bias assessed for the included literature.

The quality of the included studies was evaluated using the Cochrane risk of the bias assessment tool. The risk of bias assessment for the included studies is shown in Fig. 2, Fig. 3.

### Meta-analysis

#### Heart weight/body weight (HW/BW)

**Fig. 4:**
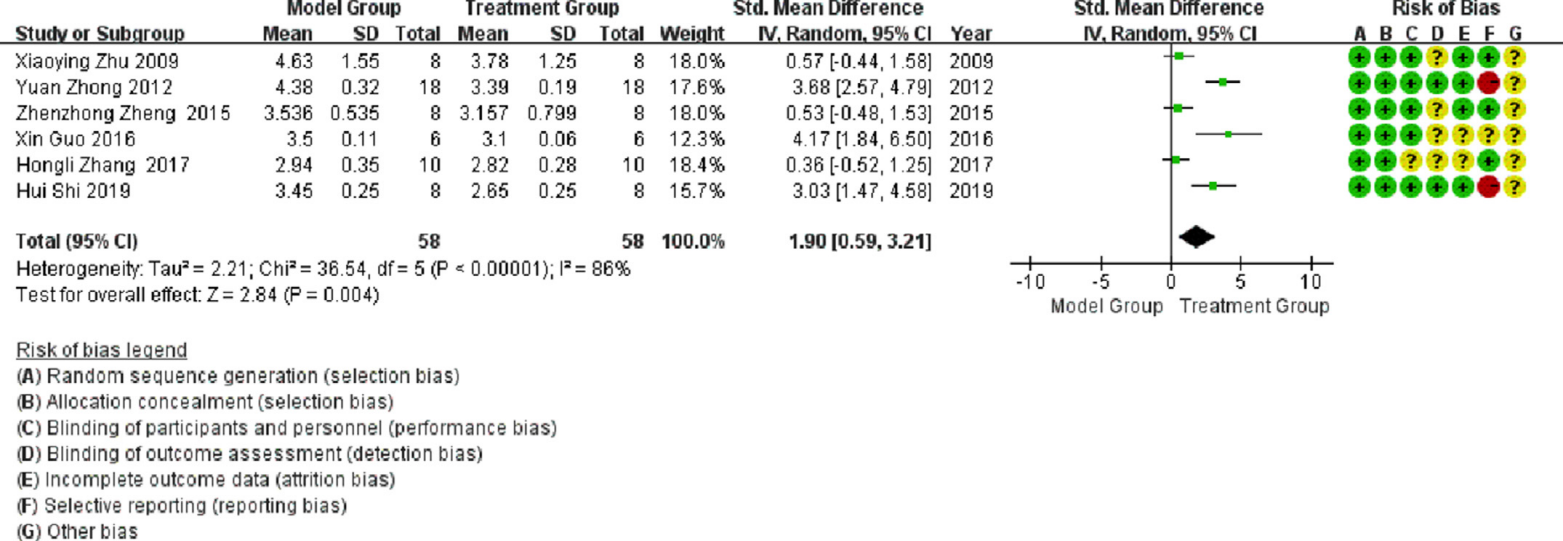
Meta-analysis of Heart Weight/Body Weight (HW/BW).

Six publications involved studies on HW/BW, with significant heterogeneity observed between them (I^2^ > 50%). Meta-analysis using a random-effects model showed that the HW/BW ratio in the treatment group was significantly lower than that in the model group (SMD = 1.9; 95% CI between 0.59 and 3.21; p < 0.05; see Fig. 4).

#### Left ventricular systolic pressure

**Fig. 5:**
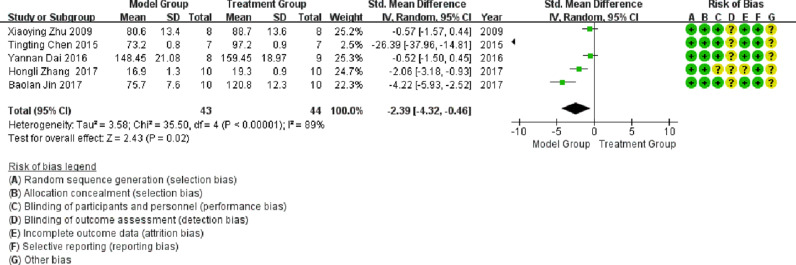
Meta-analysis of the Left Ventricular Systolic Pressure (LVSP).

Five publications involved studies on LVSP, with significant heterogeneity observed between them (I^2^ > 50%). Meta-analysis using a random-effects model showed that LVSP in the treatment group was significantly higher than that in the model group (SMD = -2.39; 95% CI between -4.32 and -0.46; p < 0.05; see Fig. 5).

#### Left ventricular end-diastolic pressure (LVEDP)

**Fig. 6:**
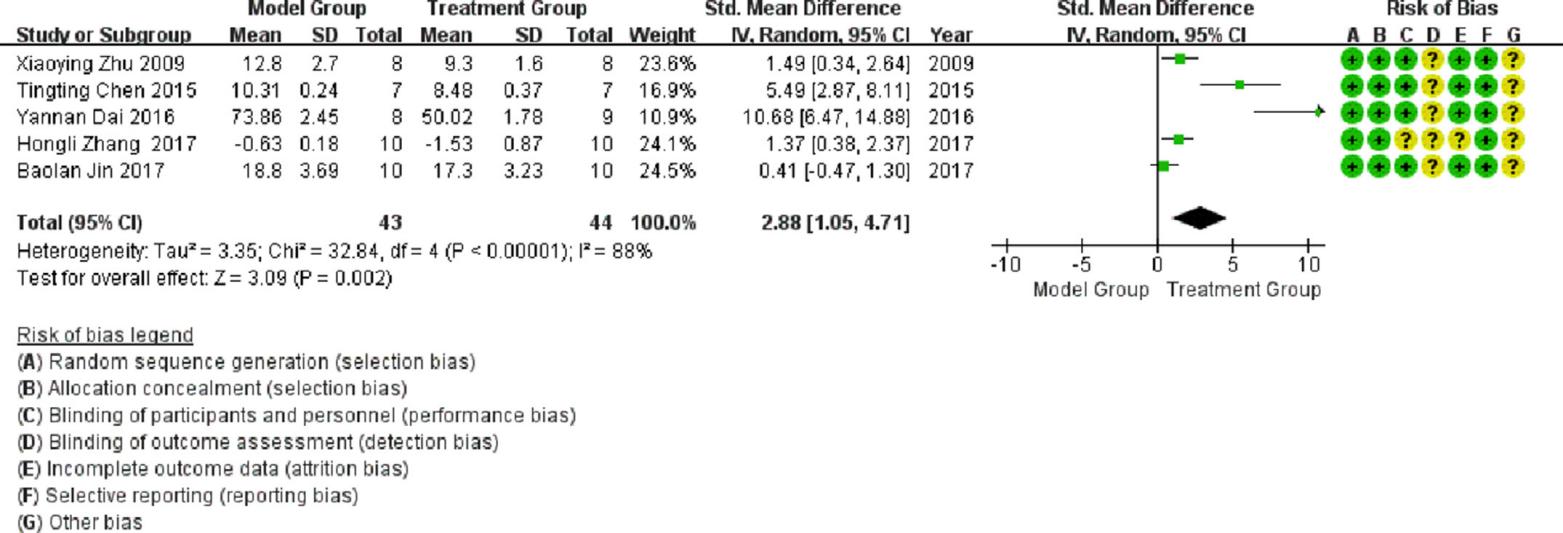
Meta-analysis of Left Ventricular End-Diastolic Pressure (LVEDP).

Five publications involved studies on LVEDP, with significant heterogeneity observed between them (I^2^ >50%). Meta-analysis using a random-effects model showed that LVEDP in the treatment group was significantly lower than LVEDP in the model group (SMD = 2.88; 95% CI between 1.05 and 4.71; p < 0.05; see Fig. 6).

#### Publication bias analysis

**Fig. 7:**
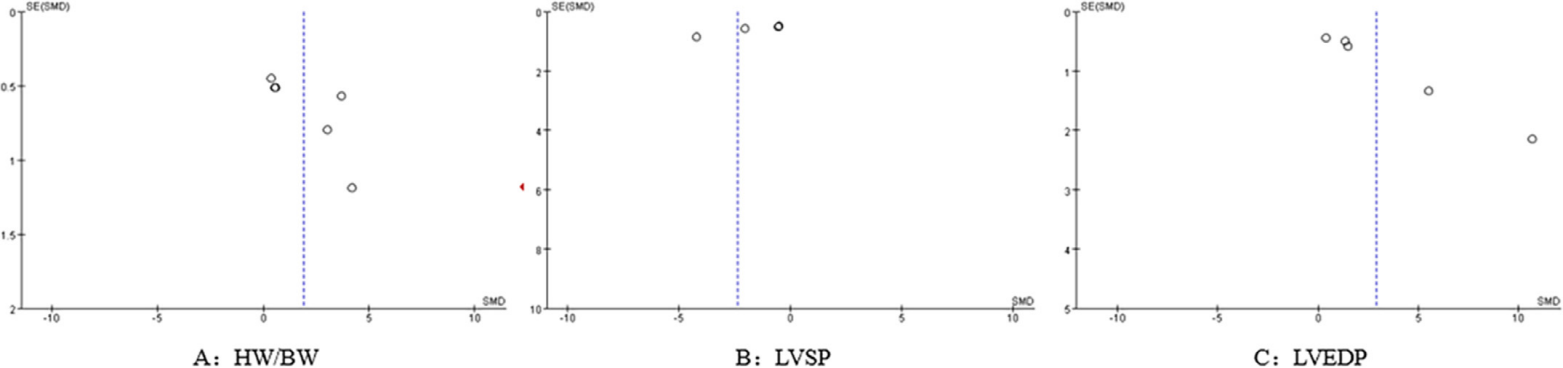
Publication bias assessment using funnel plots.

Funnel plots were generated based on HW/BW, LVSP, and LVEDP outcome indicators to assess publication bias. The results showed that the study points were asymmetrically distributed on both sides of the funnel plots, suggesting the presence of possible publication bias, as shown in Fig. 7.

#### Sensitivity analysis

**Fig. 8:**
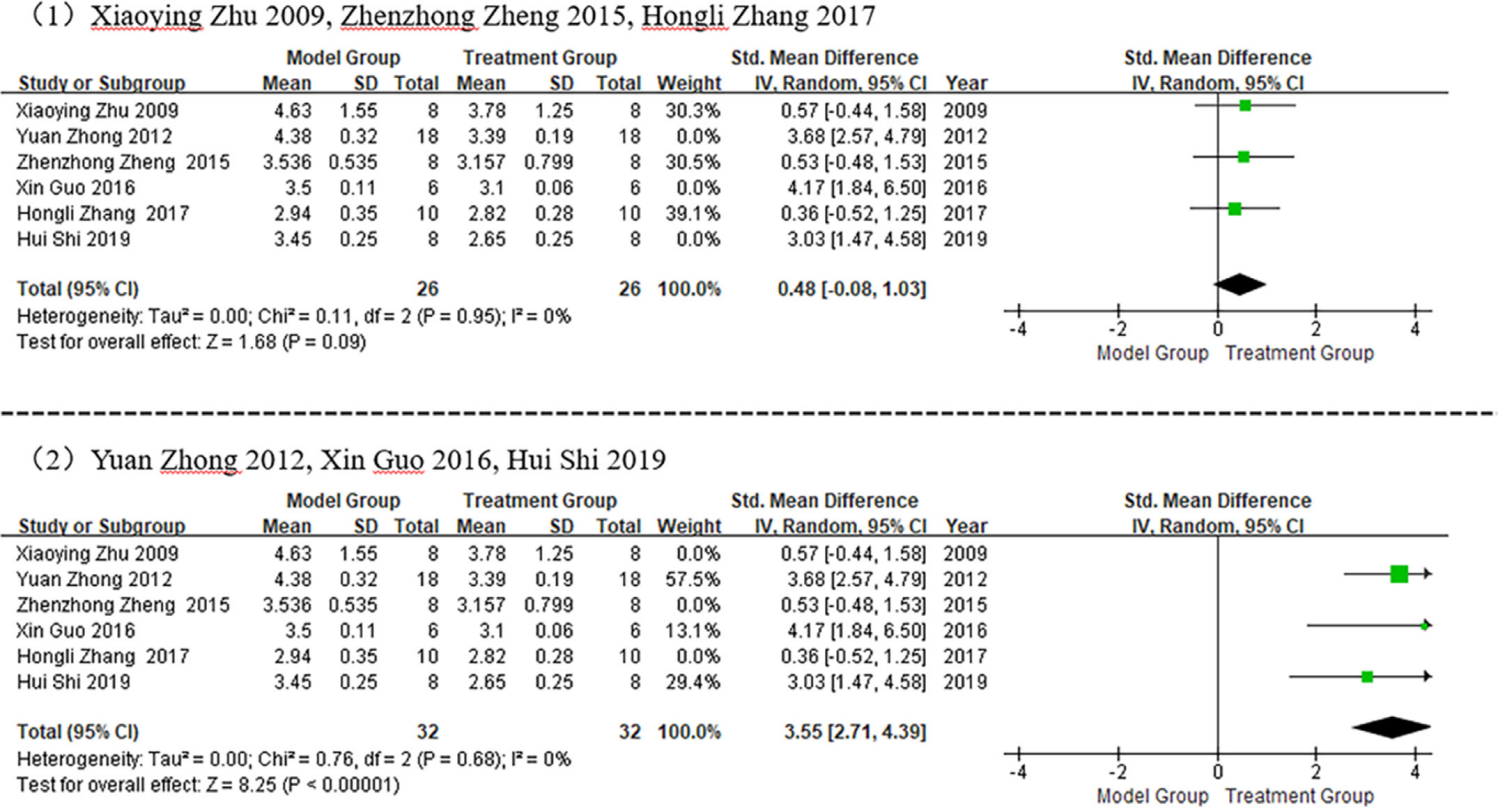
Meta-analysis after Heart Weight/Body Weight (HW/BW) grouping.

Based on the above-mentioned data analysis, it can be inferred that the resulting heterogeneity was considerable. A sensitivity analysis was performed to manually remove any group of data for statistical analysis. In a meta-analysis of studies conducted on HW/BW, the literature was divided into the following two groups: 1) Zhu Xiaoying 2009, Zheng Zhenzhong 2015, and Zhang Hongli 2017; and 2) Zhong Yuan 2012, Guo Xin 2016, and Shi Hui 2019. After grouping the literature, the heterogeneity of both groups was significantly reduced (I^2^ = 0%), as illustrated in Fig. 8, which showed that the HW/BW in the treatment group was significantly lower than that in the model group (p < 0.05).

**Fig. 9:**
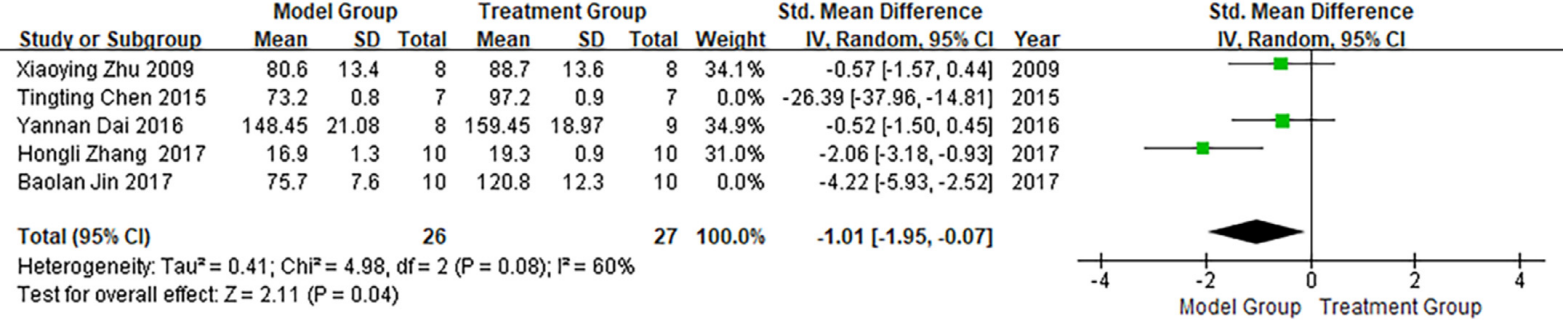
Meta-analysis of studies conducted on left ventricular systolic pressure (LVSP) after the exclusion of heterogeneous literature.

In a meta-analysis of studies conducted on LVSP, the heterogeneity was attributed to the literature published by Tina Chen in 2015 and Pauline Kim in 2017, and the heterogeneity was significantly reduced after exclusion (I^2^ = 60%), as shown in Fig. 9. As illustrated in Fig. 9, LVSP in the treatment group was significantly higher than that in the model group (p < 0.05).

**Fig. 10:**
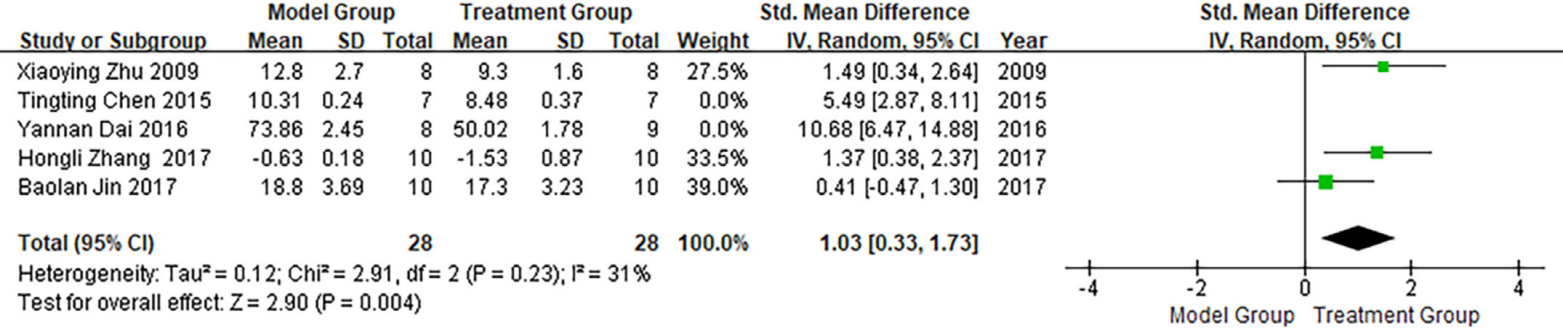
Meta-analysis of studies conducted on Left Ventricular End-Diastolic Pressure (LVEDP) after the exclusion of heterogeneous literature.

In a meta-analysis of studies conducted on LVEDP, heterogeneity was attributed to the literature published by Chen in 2015 and Dai in 2016. The heterogeneity was significantly reduced after exclusion (I^2^ = 31%), as shown in Fig. 10. As illustrated in Fig. 10, LVEDP in the treatment group was significantly lower than that in the model group (p < 0.05).

## Discussion

DCM is one of the most severe complications of diabetes mellitus and has been studied extensively in recent years. It is a specific myocardial degenerative disease caused by chronic diabetes mellitus that significantly affects the quality of life and prognosis of patients with diabetes. To counter the pathogenesis and pathophysiological characteristics of diabetic cardiomyopathy, several drugs have been investigated and proven to delay the progression of cardiomyopathy in patients with DCM (e.g., hypoglycemic drugs,^9^^,^^10^ statins,^11^ and β-blockers,^12^, ^13^, ^14^ protecting the myocardium and improving the prognosis of patients.

Toll-Like Receptors (TLRs) are an essential class of proteins involved in the establishment of non-specific immunity (innate immunity) and serve as a bridge between non-specific and specific immune responses. TLR4 expression levels were significantly higher in children with DCM compared to those in healthy children; its levels were closely associated with the size of the heart and degree of heart failure; that is, the larger the heart, the more severe the heart failure, with higher TLR4 expression levels being observed. The TLR4-NF-κB signaling pathway is one of the most critically identified inflammatory pathways associated with many diseases. Cascading responses to downstream inflammatory signals may lead to increased progression of the disease. The possible mechanisms of TLR4 involvement in DCM pathogenesis are as follows: As a transmembrane glycoprotein, TLR4 is composed of three regions: an extracellular region, a transmembrane region, and an intracellular region. Under high-glucose conditions, myosomes produce considerable amounts of lipopolysaccharide, heat shock protein 60, and free fatty acids. The extracellular region of TLR4 can recognize lipopolysaccharide and free fatty acids^15^ and bind to biomolecules, activating TLR4. Activated TLR4s, via MyD88-dependent and MyD88-independent pathways, regulate gene expression in pancreatic β-cells (insulin, PDXI, and other genes), thereby impairing insulin secretion;^16^ in contrast, they downregulate NF-κB expression, inducing the production of differentiation cytokines and chemokines,^17^ which are involved in immune and inflammatory responses, leading to myocardial damage, ventricular remodeling, myocardial systolic, and diastolic dysfunction.

## Conclusions

In this study, the inhibition of TLR4 expression levels was further demonstrated by meta-analysis, improving the degree of cardiac failure with a significant reduction in heart mass ratio (HW/BW, SMD = 1.9; 95% CI [0.59, 3.21], p = 0.004), a significant reduction in impaired LVSP (SMD = -2.39; 95% CI [- 4.32, -0.46], p = 0.02), and a significant reduction in LVEDP (SMD = 2.88; 95% CI [1.05, 4.71], p = 0.002). The TLR4 signaling pathway plays an essential role in the pathogenesis of DCM and may be a new target for DCM treatment. The development of TLR4 inhibitors may lay the foundation for the development of new therapeutic strategies.

## Authors’ contributions

Yuan J designed the study and wrote the first draft.

Yi X analyzed the data and wrote and revised the paper.

Jiang H polished the first draft and confirmed the methodology and material parts.

## Conflicts of interest

The authors declare no conflicts of interest.
